# An *Ex Vivo* Brain Slice Culture Model of Chronic Wasting Disease: Implications for Disease Pathogenesis and Therapeutic Development

**DOI:** 10.1038/s41598-020-64456-9

**Published:** 2020-05-06

**Authors:** Naveen Kondru, Sireesha Manne, Robyn Kokemuller, Justin Greenlee, M. Heather West Greenlee, Tracy Nichols, Qingzhong Kong, Vellareddy Anantharam, Arthi Kanthasamy, Patrick Halbur, Anumantha G. Kanthasamy

**Affiliations:** 1 Department of Biomedical Sciences, College of Veterinary Medicine, Iowa State University, Ames, IA USA; 2 Virus and Prion Research Unit, National Animal Disease Center, Agricultural Research Service, United States Department of Agriculture, Ames, IA USA; 3 Surveillance, Preparedness and Response Services, Veterinary Services, United States Department of Agriculture, Fort Collins, CO USA; 4 Departments of Pathology and Neurology, Case Western Reserve University, Cleveland, OH USA; 5 Veterinary Diagnostic and Production Animal Medicine, College of Veterinary Medicine, Iowa State University, Ames, IA USA

**Keywords:** Neuroscience, Diseases

## Abstract

Chronic wasting disease (CWD) is a rapidly spreading prion disease of cervids, yet antemortem diagnosis, treatment, and control remain elusive. We recently developed an organotypic slice culture assay for sensitive detection of scrapie prions using ultrasensitive prion seeding. However, this model was not established for CWD prions due to their strong transmission barrier from deer (*Odocoileus spp*) to standard laboratory mice (*Mus musculus*). Therefore, we developed and characterized the *ex vivo* brain slice culture model for CWD, using a transgenic mouse model (Tg12) that expresses the elk (*Cervus canadensis*) prion protein gene (*PRNP*). We tested for CWD infectivity in cultured slices using sensitive seeding assays such as real-time quaking-induced conversion (RT-QuIC) and protein misfolding cyclic amplification (PMCA). Slice cultures from Tg12, but not from *prnp*^*−/−*^ mice, tested positive for CWD. Slice-generated CWD prions transmitted efficiently to Tg12 mice. Furthermore, we determined the activity of anti-prion compounds and optimized a screening protocol for the infectivity of biological samples in this CWD slice culture model. Our results demonstrate that this integrated brain slice model of CWD enables the study of pathogenic mechanisms with translational implications for controlling CWD.

## Introduction

Chronic Wasting Disease (CWD) is a transmissible spongiform encephalopathy (TSE) or prion disease of cervids, with a natural incubation period in the range of 1.5–3 years^1,2^. The first clinical detection of CWD was in a captive mule deer (*Odocoileus hemionus*) in the Colorado foothills in 1967^1^. Thus far, CWD has been reported in 26 U.S. states, in 3 Canadian provinces, South Korea, Norway, and Finland^3^. CWD is a naturally occurring disease in both captive and wild cervids that spreads rapidly with horizontal^4^ and vertical transmission^5^. CWD prions have been detected in a variety of tissue samples and biological fluids such as cerebrospinal fluid (CSF) and urine^6^. The CWD agent has been reported as transmissible to a wide range of hosts by experimental inoculation^7^. For example, CWD prions can be transmitted to cattle by intracerebral (IC) inoculation with limited efficiency^8,9^. The CWD agent experimentally transmits to several other species such as ferrets^10^, raccoons^11^, small ruminants^12^, domestic cats^13,14^, and pigs^15^. In non-human primates, squirrel monkeys were highly susceptible while cynomolgus macaques appear to be resistant in one study^16–18^. More recently, prion seeding was detected in transgenic (Tg) mice that overexpress human prion protein after they were intracranially inoculated with CWD agent^19^.

Despite CWD transmission to other species, wild-type (WT) mice (*Mus musculus*) have not been shown to support a CWD prion infection^20^. All published reports so far indicate that Tg mice expressing the human prion protein gene (*PRNP*) sequence on a mouse prion null background also appear to be resistant to CWD infection^21–26^. In contrast, Tg mice expressing a cervid *prnp* sequence support efficient CWD transmission^27,28^. Kong and colleagues^22^ developed a Tg mouse model (Tg12; *prnp*^*+/−*^) expressing the elk (*Cervus canadensis*) *PRNP* sequence that has an incubation period of 118–142 days for CWD prions.

Studying CWD prions in captive cervids is both time-consuming and cost-prohibitive since the incubation period of CWD in experimental inoculations can reach 3 years in cervids and requires adequate space equipped for cervid use. Therefore, the purpose of this study was to develop and characterize an *ex vivo* CWD model to address some of these challenges to the study of CWD prions. We recently reported an integrated organotypic slice culture (OSCAR) model for studying Rocky Mountain Laboratories (RML) scrapie prions with a reduced timeframe^29^. The OSCAR model utilizes the advantages of both *ex vivo* experimentation and ultrasensitive prion detection, thereby minimizing the amount of tissue required. Furthermore, it relies on a sensitive and specific seeded amplification of infectious prions from samples, which occurs via the RT-QuIC (real-time quaking-induced conversion) assay. RT-QuIC detection of prions has become a sensitive diagnostic method for assaying samples from an array of biospecimens of either human or animal origin^6,30–35^. RT-QuIC has been recently adopted as a promising approach for the ultrasensitive detection of misfolded alpha-synuclein protein in Parkinson’s disease using human CSF, brain and submandibular salivary gland samples^36,37^. This assay has also been applied in CWD diagnosis and the evaluation of CWD therapeutics^38–40^. RAMALT (recto-anal mucosa-associated lymphoid tissue) samples have been particularly useful for pre-symptomatic detection of CWD in cervids^6,30,41,42^. Brain slices maintain a 3D environment with the integration of different cell types in the brain such as neurons, astrocytes, microglia, and oligodendrocytes. The slice model has the unique capability of enabling the testing of compounds within a system with active cellular processes involving prions, while RT-QuIC is an *in vitro* assay that requires recombinant PrP interaction. Therefore, we hypothesized that a brain slice culture model for CWD prions would facilitate the study of their biology on a shorter, more reasonable timescale. In this study, we developed and characterized brain slice cultures for the *ex vivo* examination of CWD prions. These slice cultures support CWD prion replication, and the kinetics of the prion seeding were quantified at different time points. Furthermore, we demonstrate that our CWD brain slice culture model is well suited for testing the anti-prion activity of pharmacological compounds and for detecting CWD prions sampled from RAMALT tissues.

## Results

### Brain slice cultures from cervidized mice support CWD infection

**Figure 1:**
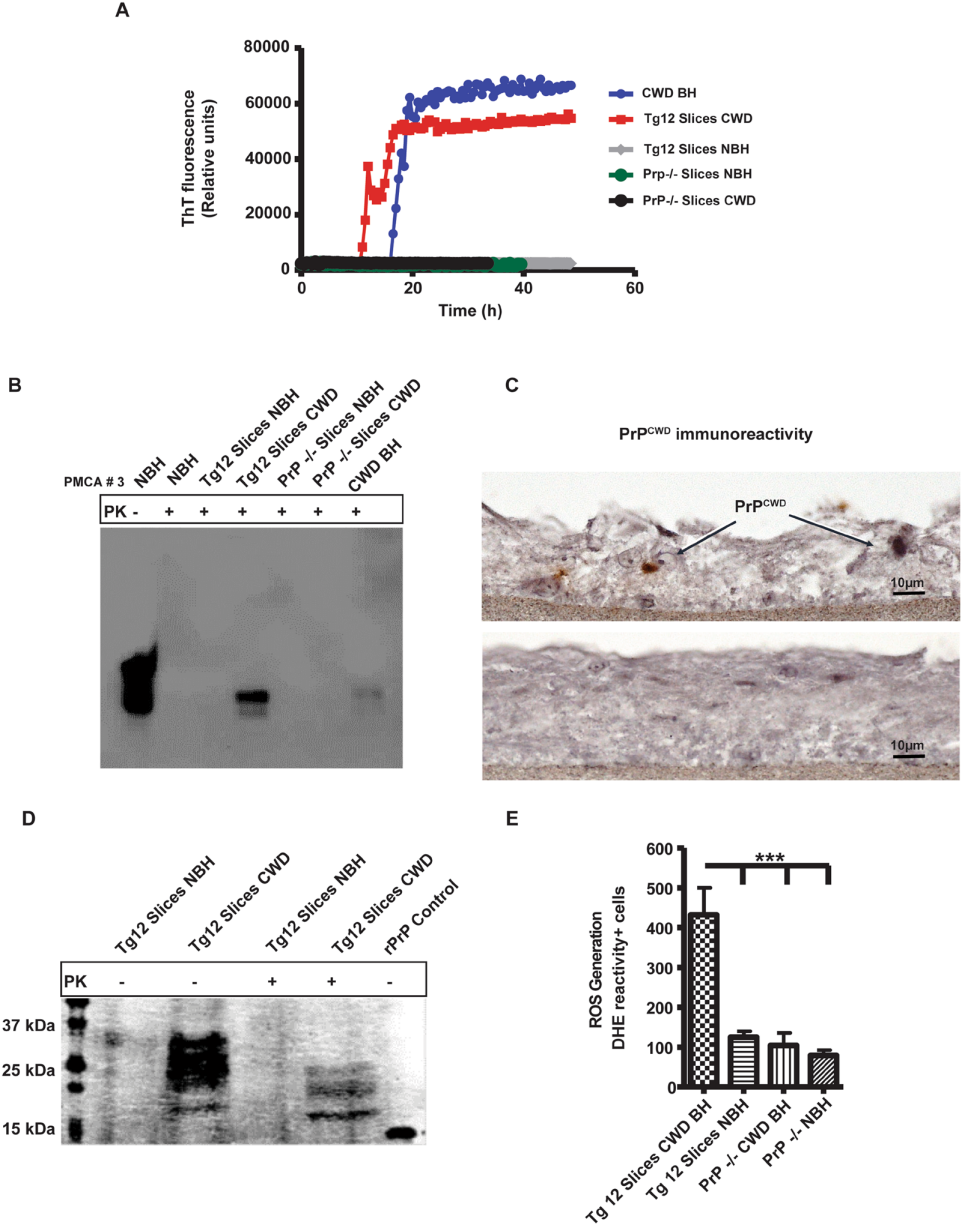
*In vitro* seeded amplification of CWD prions from organotypic slice cultures using the RT-QuIC assay and PMCA. (**A**) RT-QuIC seeding curves showing that slice cultures from Tg12 mice (*prnp*^*+/−*^) efficiently seed the RT-QuIC assay producing strong positive peaks for the reactions seeded with 5 ng of CWD-infected slice culture homogenates at 42 dpi (red trace, labeled as Tg12 Slices CWD) or brain homogenates (BH) from a CWD-infected deer (blue trace, labeled as CWD BH). Slices cultured with NBH (grey trace) and *prnp*^−/−^ slices cultured with CWD BH (black trace) or NBH (green trace) did not show seeding activity during the reaction period. Each trace is a combined average of 3 technical replicates and 2 biological replicates from a pool of 7–10 slices. (**B**) A representative full-length Western blot from the third of three serial rounds of PMCA performed on BH from the CWD slice culture model. All samples were treated with PK, except NBH from Tg5037 mice (NBH PK (−) in the first lane) used as a migration (positive) control. Only the positive control and Tg12 slices inoculated with CWD deer BH show immunoreactivity. (**C**) Representative PrP^CWD^ immunohistochemistry (6H4 antibody) images showing a cross-section of 35-dpi slices. Slices from Tg12 mice inoculated with CWD deer BH showed immunoreactivity (top image), whereas NBH did not (bottom image). Scale bar, 10 µm. (**D**) Representative full-length Western blot showing the presence of PK-resistant CWD prions in Tg12, but not *prnp*^−/−^, slice culture homogenates, all probed with the anti-*prnp* mouse monoclonal antibody POM1. **(E**) DHE conversion assay showing the significantly higher generation of reactive oxygen species (ROS) for CWD BH-infected cerebellar slices when compared to NBH-treated or *prnp*^−/−^ slice cultures. Data were analyzed using one-way ANOVA; n = 4 biological replicates. Data are mean ± SEM (*p ≤ 0.05, **p < 0.01, ***P < 0.001).

Organotypic cerebellar slice cultures, prepared from 9- to 12-day-old cervidized (Tg12) or *prnp*^−/−^ pups as described in the methods section, were exposed to CWD prion-infected brain homogenate (BH) or normal brain homogenate (NBH) as infected control and maintained in cultures for up to 52 days post-inoculation (dpi). The CWD prions that accumulated in these slices were detected using the RT-QuIC assay based on their seeding ability. As expected, the positive control CWD BH inoculum efficiently seeded the conversion of rPrP (Fig. 1A, blue trace). Importantly, brain slices from Tg12 mice exposed to CWD prions also demonstrated seeding activity at 42 dpi (Fig. 1A, red trace). However, the brain slices from Tg12 mice treated with NBH did not seed the RT-QuIC reactions (Fig. 1A, gray trace). Prions were amplified only in slices from Tg12 pups infected with BH from CWD-infected deer, not in *prnp*^−/−^ slices (Fig. 1A, green and black traces), demonstrating the specificity of this slice culture model. Prion seeding activity from cerebellar slices was also observed with serial protein misfolding cyclic amplification (sPMCA) (Fig. 1B). The sPMCA analysis confirmed that only cervidized slices were able to maintain the CWD prions and develop PK-resistant bands on Western blots. Brain slices from *prnp*^−/−^ mice treated with NBH or infected BH did not demonstrate PK-resistant material on their Western blots (Fig. 1B).

Next, IHC was done on the slice cultures to demonstrate PrP^CWD^ accumulation in slice culture. Distinct PrP^CWD^ immunoreactivity was observed from slices infected with CWD prions when tested at 35 dpi but not in the NBH-treated control group (Fig. 1C). Hematoxylin and eosin (H&E) staining images of slice cultures demonstrate the preservation of tissue architecture (Supplementary Fig. 1). Immunohistochemical staining of slice cultures was also performed using NeuN and calbindin staining to demonstrate the preservation of neurons including Purkinje cells (Supplementary Fig. 2). Lysates prepared from the slices were then digested with PK for determining the presence of PK-resistant prions by Western blot (Fig. 1D). Slices infected with CWD prions demonstrated the distinct pattern of di-, mono-, and non-glycosylated PK-resistant bands of PrP^CWD^ on Western blots, with bands shifted lower than those from slices not treated with PK. This indicates a lower molecular weight of fragments after treating with PK, along with the characteristic glycosylation banding pattern of misfolded prions (Fig. 1D). To determine if CWD prions induce oxidative stress in slice cultures, we measured the reactive oxygen species (ROS) production from the slice cultures using the dihydroethidium (DHE) conversion assay (Fig. 1E). DHE is a live-cell-permeable stain that reacts with ROS and results in a fluorescence product that can be imaged by microscopy. The *prnp*^*+/−*^ slices infected with CWD demonstrated significantly higher ROS reactivity when compared to controls. Taken together, these results suggest that the CWD prions can readily infect and propagate in slice cultures when the elk PrP is expressed and that our CWD slice culture model displays key features of CWD pathology.

### Kinetics of CWD prion propagation in *ex vivo* cerebellar slice cultures

**Figure 2:**
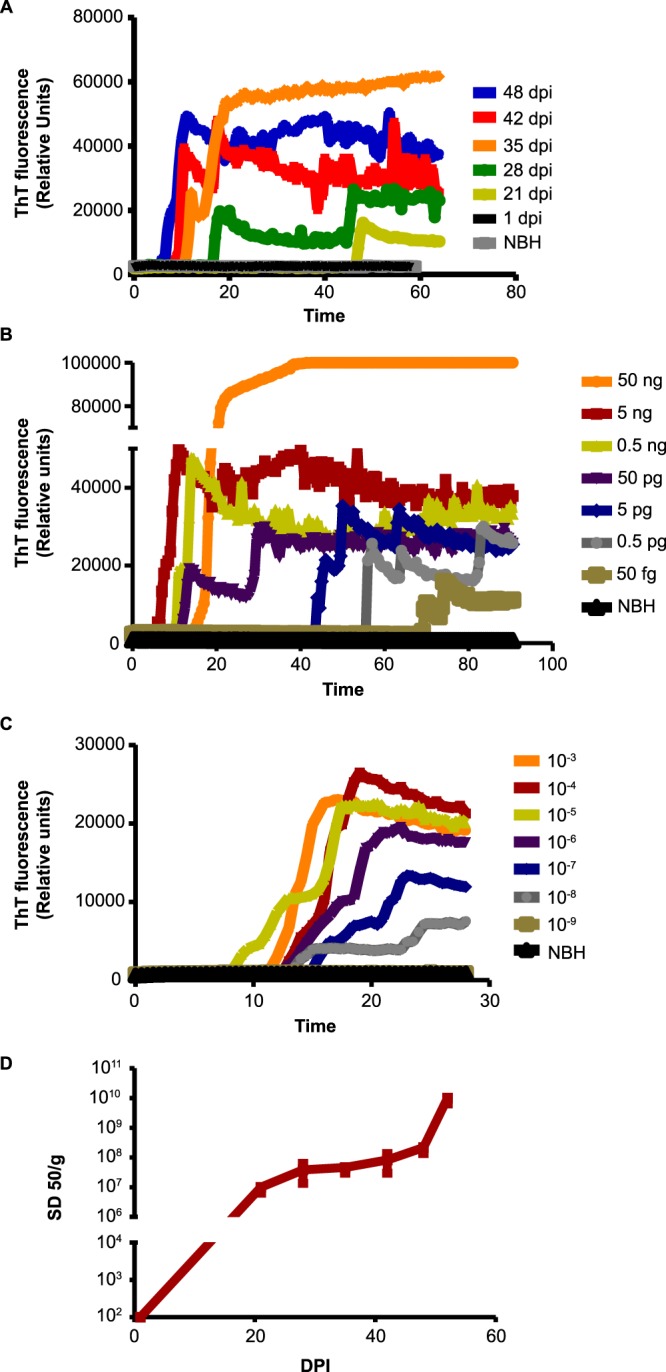
Seeding profile of CWD prions from organotypic slice cultures. (**A–C**) Representative traces from RT-QuIC assay of slices exposed to brain homogenates from normal (NBH) or CWD-infected deer and cultured up to 52 dpi. Each trace comprises 0.5-h time points represented as an average ThT fluorescence from n = 3–4 replicate wells. (**A**) Seeding activity in CWD-infected slices accelerated rapidly after 21 dpi (n = 3). (**B**) Lysates prepared from 52-dpi slices were serially diluted 10-fold, ranging from 50 fg to 50 ng. The reactions (n = 4), consisting of 5 µL of these dilutions as seed, revealed optimal seeding doses ranging between 50 pg to 50 ng. (**C**) Endpoint 10-fold serial dilution analysis (n = 4) of the CWD deer BH used as inoculum. **(D**) Quantification of seeding activity from different time points in the CWD slice culture model. The median seeding dose (SD_50_) is defined as the amount giving sufficiently enhanced ThT fluorescence in half of the replicate wells. The Spearman-Kärber estimate of the SD_50_, plotted against days post-inoculation of the slice culture, shows prion titers progressively increase to a maximum at 52 dpi.

To determine the potential application of our CWD slice culture as an *ex vivo* bioassay model for monitoring CWD infection, we exposed slice cultures to either NBH or BH from CWD-infected deer and monitored the kinetics of CWD prion propagation for 1–52 days. The seeding activity from each time point tested (1, 21, 28, 35, 42, 48 and 52 dpi) was examined by RT-QuIC. The seeding ability of CWD-infected *prnp*^*+/−*^ slice cultures substantially increased with time after 21 dpi (Fig. 2A). In contrast, NBH slice cultures did not demonstrate seeding activity at any time point (Fig. 2A). To further delineate the seeding dose (SD) required for RT-QuIC, we serially diluted (10-fold increments from 50 fg to 50 ng) homogenates prepared from slice cultures at their terminal infective point, 52 dpi. Seeding activities were detected at each dilution $\ge$50 pg as defined by a time-to-reach threshold of <40 h (Fig. 2B). To compare the seeding activity of prions in slice cultures inoculated with CWD BH, we tested the seeding kinetics of different 10-fold dilutions of a CWD BH in RT-QuIC (Fig. 2C). The relative seeding of the slice cultures is comparable to the CWD BH used for inoculation (Fig. 2B,C). RT-QuIC was able to detect even femtogram concentrations of CWD prion in the slice culture, demonstrating the ultra-sensitivity and the advantages of combining the methods of CWD slice culture and RT-QuIC. Furthermore, the time-dependent increase in the accumulation of prions is well highlighted by employing the Spearman-Kärber method to derive the SD_50_ (median seeding activity, 50% of replicate wells) values (Fig. 2D), which continued to increase up to 52 dpi. Taken together, these results demonstrate that slices accumulated detectable levels of prions after 21 dpi, and the seeding titers increased over time, displaying seeding kinetics comparable to BHs from terminally ill CWD-infected deer.

### Slice cultures as models for transmission assays and screening therapeutics

**Figure 3:**
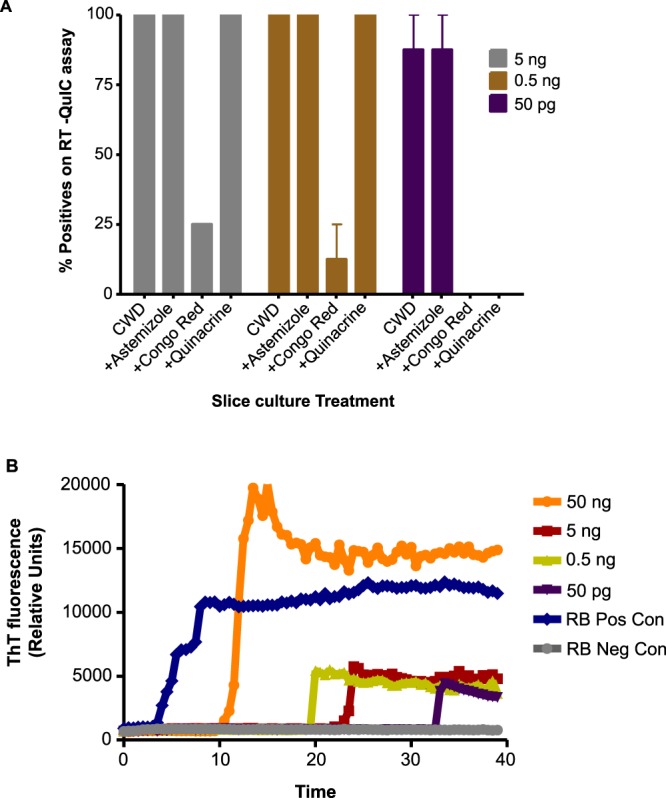
The anti-prion activity of compounds and the capture of CWD prions from biological samples using slice cultures. (**A**) The anti-prion activity of the compounds astemizole, Congo red, and quinacrine was evaluated against a CWD-infected control and 5 ng, 0.5 ng, 50 pg serial log dilutions. Inhibitory activity depended on the seed dilution, except for astemizole, which had no apparent effect on the seeding activity of CWD prion-infected slice cultures. (**B**) RT-QuIC assay showing transmission of CWD prions from infected recto-anal mucosa-associated lymphoid tissue (RAMALT) samples to the slices cultured with RAMALT homogenates for 21 days when tested at log-fold dilutions of 50 ng to 50 pg. A CWD-infected RAMALT homogenate was used as a positive control, while a healthy RAMALT control homogenate served as a negative control. Each RT-QuIC trace represents the average fluorescence reading from 3 technical replicates of slice cultures.

Next, we examined the utility of CWD slice cultures for transmission assays and for screening anti-prion compounds. Slice cultures were treated with three anti-prion compounds, including astemizole, Congo red, and quinacrine, and the seeding activity of prions was titrated at various seeding doses (Fig. 3A). Congo red significantly reduced the RT-QuIC amplification kinetics of misfolded prions, as evident at the 5-ng and 0.5-ng seeding doses of CWD-infected slices. Likewise, at a seeding dose of 50 pg, Congo red completely inhibited the seeding activity (Fig. 3A). However, astemizole failed to inhibit the seeding activity at any tested seeding dose, and quinacrine only inhibited the seeding activity at the 50-pg seeding dose (Fig. 3A). Next, we used RAMALT samples to test the utility of slice cultures to amplify prions from clinical samples. RAMALT samples were homogenized and applied to slice culture media after it had been cultured in normal media for 7 days. After a 24-h inoculation, the media was replaced with fresh media. After culturing for another 3 weeks, the slices were harvested, and lysates of different concentrations (50 pg to 50 ng) were used to assess the seeding ability of these slice cultures relative to known CWD-positive RAMALT samples (Fig. 3B). Prion amplification kinetics suggests that seeding activity was present in these slices. Slices exposed to 50 ng of a CWD-infected RAMALT sample had the highest seeding ability when compared to RAMALT control slices, followed by other slice seeding doses (Fig. 3B). Collectively, these results suggest that this slice culture model of CWD is suitable for screening anti-prion compounds and for detecting CWD prions sampled from RAMALT tissues.

### Infectivity of prions generated in slice culture models

**Figure 4:**
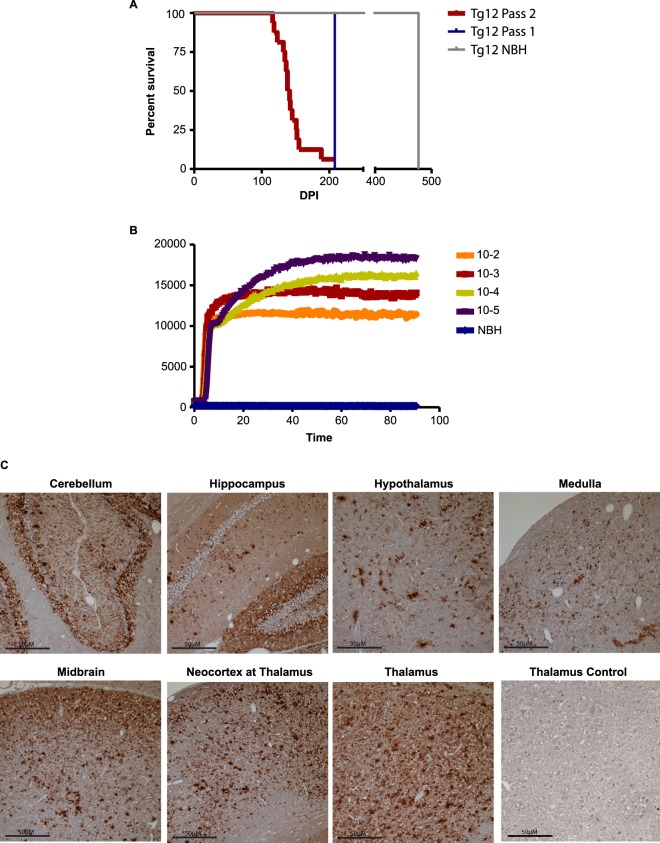
Mouse bioassay of CWD prions from slice cultures. (**A**) Survival analysis of Tg12 mice intracranially inoculated with 5-µL lysates from homogenized CWD-inoculated brain slices during passage 1 (Pass 1) showing an incubation period of 208 days. Mice inoculated with NBH slices tested negative for PrP^CWD^ at the end of the experiment. When Tg12 mice (Pass 2) were similarly injected with 5-µL of inoculum from Pass 1 mouse brains, the mean incubation period was reduced to 139.5 dpi. (**B**) RT-QuIC assay reaction traces (n = 4 replicates) showing efficient CWD prion transmission to the brains of mice in passage 1. (**C**) Immunohistochemistry of paraffin-embedded tissue sections showing neuropathology and CWD prion plaques in multiple brain regions of mice from Pass 2. No immunoreactivity is present in a representative section of the thalamus from a negative control Tg12 mouse. Plaques were more abundant in the granular layer of the cerebellum and hippocampus. A diffuse pattern was observed in the hypothalamus and medulla while a mixed pattern manifested in the midbrain and thalamus. Scale bars: 50 μm.

To test whether prions generated in slice cultures propagate prion infectivity, we intracranially inoculated Tg12 mice with homogenates prepared from CWD-exposed slice cultures (representing passage 1). These mice died after an average incubation period of 208 days with clinical signs characteristic of prion infection. Mice that were inoculated with NBH-treated brain slice homogenates remained asymptomatic until the end of the experiment at 477 days. BHs from passage-1 mice were subsequently passaged by intracerebral inoculation to other Tg12 mice (passage #2), which were then monitored for clinical signs. The second passage showed earlier clinical signs and had a median incubation period of 139.5 days (Fig. 4A). When the BHs from passage-1 mice were tested in serial 10-fold dilutions using RT-QuIC, a potent seeding activity was observed in all dilutions tested, indicating efficient transmission (Fig. 4B). This incubation period is most closely aligned with the mean incubation period in Tg12 mice inoculated with CWD deer BH as reported previously^22^. Immunohistochemistry (IHC) reveals that PrP^CWD^ immunoreactivity was observed in all the brain regions inspected from passage-1 mice infected using inoculum from CWD prion-positive slice cultures (Fig. 4C). The granular layer of the cerebellum and hippocampus manifested abundant PrP^CWD^ plaques, while a diffuse pattern of plaque deposition was observed in the hypothalamus. A mixed pattern of plaque deposition was manifested in the midbrain and thalamus. Taken together, these results suggest that prions developed in slice cultures can successfully infect the Tg12 mouse model.

## Discussion

CWD is highly transmissible and rapidly spreading to dozens of states in the USA, particularly in the last few years^43,44^. Associated with this trend is an increase in the shedding of CWD prions and their subsequent persistence in the environment. Despite the growing threat posed by CWD, we still lack an adequate *ex vivo* culture model to study this disease. In the current study, we developed a CWD brain slice culture model to test the utility of propagating CWD prions using this technique. We prepared slice cultures from either transgenic *prnp*−/− mice or Tg12 mice that express an elk *prnp* +/−. The slices were cultured and tested for seeding activity using RT-QuIC. Tg12 slices successfully propagated infectious CWD prions in contrast to the *prnp*^−/−^ slices, which did not show amplification. This confirms the proof-of-concept that CWD prions replicate in the presence of cervidized *prnp* expression consistent with the well-documented phenomenon that *prnp* is required for prion infectivity and propagation^45^. We further demonstrated the presence of PrP^CWD^ immunoreactivity in slices from Tg12, but not in *prnp*^−/−^ mice, suggesting the presence of PK-resistant prions in the slice cultures. DHE is a specific adduct of ROS and forms a fluorescent product, 2-hydroxyethidium, that can be captured using fluorescence when an ROS is generated^46^. In our study, CWD prions induced oxidative stress in slices, as evidenced by significantly higher DHE reactivity when compared to all controls (CWD BH-treated *prnp*−/− slices and NBH-treated *prnp* +/− slices). This novel *ex vivo* CWD brain slice model allowed us to demonstrate that oxidative stress underlies CWD pathogenesis. H&E staining and immunohistochemistry of the slice cultures demonstrate the relative preservation of cell types and overall tissue architecture (Supplementary Figs. 1 & 2). We cannot completely exclude the possible existence of some necrosis in the tissues because it was technically challenging to process slice cultures attached with membrane. Additionally, we and others have previously shown that the cultured slices are viable for several weeks^47–50^.

We also determined the seeding ability and median seeding dose of CWD prions using PMCA and RT-QuIC. In agreement with other CWD prion studies showing differential tropism when their distribution is examined across multiple brain regions, we noted substantial cerebellar and hippocampal depositions with less consistent accumulations in the midbrain, thalamus, and hypothalamus. This pattern resembles captive mule deer brains, which have PrP^CWD^ deposition mostly in the medulla and basal ganglia, with only mild cerebellar involvement, and lowest accumulations in the cerebral cortex^51^. Characterizing the seeding activity (a relative measure of infective prions) based on serial log dilutions at different time points revealed a steady increase in prion titers, suggesting the time-dependent amplification of CWD prions in slice cultures. The seeding activity in slice culture peaked at 52 dpi, which is comparable to a terminally infected CWD BH. Overall, the accumulation of prions in slice cultures showed a time-dependent increase.

Different strains of CWD prions have been identified that possess distinct conformations and biological properties^52,53^. For instance, even though quinacrine has been extensively reported to have anti-prion activity against the RML scrapie strain, its application can enhance the propagation of CWD prions^54^. Therefore, we determined the activity of quinacrine and two other anti-prion compounds, astemizole and Congo red, in our CWD prion slice culture model since their activity had been tested previously in slice cultures exposed to RML scrapie prions^29,55^. Based on reactions seeded in the RT-QuIC assay, astemizole had no effect on CWD prion titers at any of the seed concentrations tested. In contrast, Congo red reduced CWD prion titers in all the RT-QuIC reactions seeded, including a 75% reduction in seeding capacity when 5 ng or 0.5 ng of seed was used and a 100% reduction in positive wells with 50 pg of seed. On the other hand, quinacrine did not reduce the percentage of positive RT-QuIC reactions seeded with either 5 or 0.5 ng of CWD prions. This effect is similar to quinacrine’s effect of promoting CWD prions as reported in Tg mice^56^. However, quinacrine did inhibit 50-pg seeded conversions, suggesting a seed concentration-dependent amplification profile.

Our slice culture model successfully captured the prion infectivity sourced from RAMALT tissues biopsied from a CWD-positive deer^57^. Although further testing with more RAMALT biopsied samples is warranted, our results suggest that the CWD brain slice culture model may be suitable for experimental testing of a variety of biological tissues that are known to harbor prions, especially given recent reports that CWD prions can be detected in peripheral lymph nodes, CSF, and saliva^30,58–62^. It is noteworthy that residual challenge material from infected slice cultures did not contribute to the observed RT-QuIC seeding activity as we did not detect RT-QuIC amplification from residual inoculum used to infect the PrP−/− slice cultures (Fig. 1A). Other Tg mouse models (GtE226 and GtQ226) were recently developed by a gene-targeting approach to study prion conformations/strains and appear to be advantageous, although they have yet to be tested as slice culture models^63^. Overall, we believe our cervidized *ex vivo* mouse model of CWD, aided by the ultra-sensitive detection of infectivity afforded by the broader dynamic range of evolving seeding assays, will help elucidate CWD prion biology in the context of compound screening among other relevant analytical applications. In summary, our study demonstrates that coupling a CWD slice culture model with RT-QuIC can be adopted for CWD bioassay, transmission rate monitoring, and therapeutic development.

## Methods

### Ethics statement

Experiments were performed according to the *Guide for the Care and Use of Laboratory Animals*. All experiments involving animals were done with the approval of the Institutional Animal Care and Use Committee (IACUC) at Iowa State University and the National Animal Disease Center under protocol numbers 3985 and ARS-2730.

### Slice culture assays

Organotypic cerebellar slice cultures were done as reported previously^47,64^. In brief, organotypic cerebellar slices were prepared from 10- to 12-day-old Tg mouse pups from either Tg12 or prion gene knockout (*prnp*^−/−^) mice from the same litter. Since the genotype is unknown at the time of culture preparation, tail samples were collected at dissection and frozen until genotyping was performed. The cerebellum was dissected out from the whole brain and prepared for the collection of 350-µm sagittal plane slices using a vibrating microtome (Compresstome VF-300, Precisionary Instruments). During slicing, the cerebellum was held in place inside a specimen holding tube filled with warmed 2% low-melting agarose that was rapidly solidified with an ice-cold chilling block. The tube was then secured into the vibratome’s slicing bath filled with ice-cold Gey’s balanced salt solution along with kynurenic acid (GBSSK) where the agarose-embedded cerebellum was sliced using a medium speed setting, and collected slices were transferred to a 6-well plate filled with ice-cold GBSSK. Next, a pool of 20–30 sagittal cerebellar slices were incubated for 1 h at 4 °C with BHs that had been collected from the obex region of either uninfected controls or CWD-infected deer brainstems. Having been diluted in the GBSSK solution, each BH treatment had a final concentration of 100 µg/mL. After treatment, slices were washed twice with fresh, ice-cold GBSSK and then transferred onto membrane inserts preloaded in 6-well plates. The residual buffer was removed, and 1 mL of fresh media was added to the basolateral compartment of the plate, avoiding any air bubbles at the base of the membrane. The slices were maintained in a standard cell culture incubator with 5% CO_2_ at 37 °C. The slice culture media was replaced on alternate days with pre-warmed media. Tg12 slices infected with CWD BH were cultured for various periods and harvested after 1, 21, 28, 35, 42, and 48 dpi. After the desired endpoint, cultured slices were twice-washed in 1 mL of ice-cold phosphate-buffered saline (PBS) and harvested in 0.5 mL of PBS and the tissues were pelleted by centrifugation at 500 × g for 5 min. The pellets were stored at −80 °C until analysis. Prior to RT-QuIC, pooled slices were homogenized in 100 µL of PBS using repeated pipetting, vortexed and sonicated at 90% amplitude in a cup sonicator for 5 min. The homogenates were adjusted to a final concentration of 1 mg/mL based on absorbance readings at 280 nm wavelength and serially diluted 10-fold for testing. For IHC, slices were washed once in ice-cold PBS, and 4% paraformaldehyde was added for overnight treatment at 4 °C.

The activity of anti-prion compounds was tested on slices as described previously^64,65^. The drug doses were selected based on previous studies^64,65^. The slices were treated with a final concentration of astemizole (2 µM), Congo red (1 µg mL^−1^), or quinacrine (1 µM) in the culture media prior to media change. The treatments were started on day 15 post-inoculation and maintained by the addition of fresh compound-supplemented media on every alternate day for an additional 35 days. After the endpoint, slices were washed twice in 1 mL of PBS and harvested as described above. For testing RAMALT samples, the RAMALT homogenate was obtained from a CWD-infected elk and filtered through a 0.22-µm filter. This homogenate was diluted to obtain a 100-µg/mL final concentration in the slice culture media and incubated with 7-day-old slices for 24 h. Later, the media was replaced with fresh media and cultured for an additional 28 days in regular media. The slices were washed twice in PBS, harvested and tested as described above.

### Animal inoculations

The slice culture lysates were intracranially inoculated into 6- to 8-week-old Tg12 mice expressing the 132 M elk *PRNP* gene. The lysates were prepared from a pool of 6–7 slices washed twice with 1 mL of ice-cold PBS, pelleted and frozen at -80 °C until injection. The pellets were homogenized in 60 µL of PBS using a fine needle syringe to make a suspension. This homogenate was inoculated intracranially (30 µL/mouse) as previously described^66,67^. Brains were collected after mice were euthanized either when clinical signs developed or at the end of the experiment (477 days). For IHC evaluation, brains were fixed in 10% neutral-buffered formalin and embedded in paraffin wax. After sectioning, 4-µm sections were affixed to positively charged slides for immunolabeling with the prion monoclonal antibody 6H4. These mice injected with slice cultures represent Passage 1 (P1) and the mice inoculated with BH from P1 mice represent P2. P2 mice were inoculated with 30 µL of 10% CWD-infected BH obtained from P1 mice. The tissues were collected as described above.

### Western blot analysis

Proteinase K (PK) digestion and Western blot analysis were done according to previously published protocols^47,68–70^. In brief, after washing the slices twice with ice-cold PBS, they were harvested by scraping them off the membrane insert and triturating with PBS. Equal amounts of protein were loaded on 15% sodium dodecyl sulfate-polyacrylamide gel electrophoresis (SDS-PAGE) gels after determining the concentrations using a Bradford assay. Proteins separated using electrophoresis were transferred onto a nitrocellulose membrane. Membranes were blocked with LI-COR blocking buffer (LBB) for 1 h. They were then incubated with the primary antibodies overnight, followed by a secondary antibody incubation using an Alexa Fluor-conjugated anti-mouse antibody. For PK digestion experiments, 25 µg/mL of PK was used to digest 200 µg of protein for 30 min at 37 °C followed by boiling samples for 10 min. These samples were used to separate the proteins with electrophoresis after transferring the proteins onto nitrocellulose membranes conjugated with the POM1 *prnp* antibody at 1:5000 concentration (Prionatis AG) overnight at 4 °C. Secondary antibodies were added after triple-washing with 0.1% Tween in 1X PBS. Images were captured with an Odyssey IR imaging system using the manufacturer’s instructions.

### Immunohistochemistry

Paraffin-embedded tissues were analyzed using IHC for the detection of PrP^CWD^. In brief, brain slices were deparaffinized and rehydrated prior to a 20-min autoclave cycle in an antigen retrieval solution (Dako Target Retrieval Solution, Dako Corp., Carpinteria, CA). Tissues were then blocked with 3% hydrogen peroxide and Background Buster (Innovex Biosciences Inc., Richmond, CA) for 20 min each at room temperature (RT). The primary antibody, anti-*prnp* mAb 6H4 (1:200; Prionics, Zurich, CH), at a concentration of 0.9 mg/mL, was diluted with Dako antibody diluent and incubated for 96 h at 4 °C. Mouse brain tissues were incubated for 15 min in 98% formic acid prior to a 30-min autoclave cycle. Tissues were then incubated with primary antibody overnight at 4 °C (6C2; CVI-WUR, Lelystad, NL). The secondary antibody, Dako EnVision^+^ Dual Link System HRP, was incubated at RT for 45 min. Tissues were then washed with Tris-buffered saline and 0.05% Tween 20 (TBS-T). A DAB Peroxidase Substrate kit (Vector Laboratories Inc., Burlingame, CA) was used to stain the slides for 3 min followed by counterstaining with H&E (Leica Microsystems Inc., Wetzlar, DE) for 2 min. Tissues were then dehydrated and coverslipped. Images for the figures were captured using a Nikon DS camera on a Nikon Eclipse 55i microscope. Slice cultures were also probed with fluorescent antibodies (Alexa Fluor 555 or 488 conjugated secondary antibodies) as described previously^47^.

### Recombinant prion protein purification for RT-QuIC substrate

Truncated Syrian hamster (*Mesocricetus auratus*) recombinant prion protein (SHrPrP) (amino acid residues 90–231) was expressed and purified as per previously published protocols^37,47,71^. In brief, SHrPrP (90–231) was expressed in the Rosetta (DE3) strain of *E. coli* with kanamycin and chloramphenicol as antibiotics of selection. Overnight autoinduction supplements were added to express the protein. Since SHrPrP is localized to inclusion bodies, BugBuster (Millipore) was used to lyse the bacterial cells and isolate inclusion bodies. These inclusion bodies were denatured in 8 M guanidine hydrochloride (GuHCl) for about 1 h on a rotor. The nickel-nitrilotriacetic acid (Ni-NTA Superflow) resin was equilibrated with 6 M GuHCl for 15 min on a rotator. After equilibration with Ni-NTA beads, the inclusion bodies were loaded in an AKTA#XK26 column and purified using affinity chromatography on a Bio-Rad Duo Flow or AKTA pure system at 4 °C. On-column refolding was done from 100% denaturing to 100% refolding conditions. Elution of recombinant prion protein (rPrP) was due to imidazole and rPrP competitively binding to Ni-NTA resin. After elution, peak fractions of the pure rPrP were collected in a pre-chilled dialysis buffer (10 mM sodium phosphate buffer, pH 5.8) and dialyzed in two exchanges of 3.6 L of dialysis buffer. Finally, pure rPrP was filtered and the concentration of protein was determined based on absorbance at 280 nm. The absorbance at 280 nm was multiplied by the extinction coefficient to get the final rPrP concentration in mg/mL. Protein was aliquotted and stored at -80 °C until being used for experiments. Each batch of purified protein was tested for quality using Western blot analysis and test plates before being subjected to a seeding assay.

### Real-time quaking-induced conversion (RT-QuIC) assay

RT-QuIC analysis was done as per previously published protocols^33,42,47,72–74^. Briefly, the reaction mixture consisted of 350 mM NaCl, 0.1 M EDTA, 10 µM Thioflavin T (ThT), and 0.1 mg/mL rPrP, along with 0.0025% SDS in PBS. After adjusting the slice culture samples to 1 mg/mL based on absorbance 280 nm, 10-fold serial dilutions were made in PBS. Unless specified, a 4-log dilution was used to test samples in RT-QuIC assay. In each well of a 96-well plate, 95 µL of the reaction mixture was loaded with 5 µL of seed sourced from either CWD or NBH from deer, or from slice cultures treated with either CWD or NBH. The reaction was carried out in a sealed Nunc 96-well plate incubated at 42 °C in a plate reader with an alternating 1-min shake and rest cycle. The plates were assayed in either a Cytation 3 (BioTek) or CLARIOstar (BMG), with ThT fluorescence readings taken every 30 min at wavelengths of 450 $\pm$ 15 nm excitation and 480 $\pm$ 10 nm emission, and data analysis was performed using MARS V5.2 R8 and Gen 5 version 2.07.17 software for CLARIOstar and Cytation 3, respectively. After exporting the fluorescence readings, graphs were plotted using GraphPad Prism 7.0. A sample was determined to be positive when it crossed the fluorescence threshold, which was defined as the average mean fluorescence of control samples plus 10 standard deviations. Each sample was tested in triplicate or quadruplicate.

### Protein misfolding cyclic amplification (PMCA)

Slice culture samples were diluted to 1 mg/mL concentration in 0.5% Triton X-100 in PBS and further serially diluted to 1000-fold. The samples were blinded (to author T.N.) and subjected to PMCA as described previously^75^. In brief, the brains from 4- to 6-week-old Tg12 mice were dissected after perfusion with PBS, transferred to tubes containing PMCA buffer (PBS with 150 mM NaCl, and 4 mM EDTA) and 2.5-mm glass beads (BioSpec), and then homogenized into a 10% w/v solution using a Blue Bullet homogenizer (Next Advance) for 4 min. Next, the samples were cleared of cellular debris by centrifugation and the supernatant was removed and stored at −80 °C. Later, the lysates were subjected to three 10-fold dilutions in PBS and used as substrates to seed the PMCA reactions for three rounds. After PMCA, the amplified samples were analyzed by Western blot as described in a previous report^76^. A migration control was included using NBH from Tg5037 mice, which were engineered to express a 5-fold increase in elk PrP^c^. The characterization and bioassay of Tg5037 mice have been reported previously^77^.

### Dihydroethidium (DHE) conversion assay

To determine the reactive oxygen species (ROS) generated by CWD prions, a DHE conversion assay was performed as described previously^46^. DHE is widely used to detect ROS because of its spectral properties. DHE is internalized by live cells where it can be oxidized to a red dye and measured using 530-nm excitation and 610-nm emission wavelengths^78,79^. Adherent slices in culture at 42 dpi were washed twice in pre-warmed GBSS and incubated with 10 µg/mL DHE for 20 min in GBSS. Fluorescence microscopy imaging was performed on the slices using a 10X objective. The images were captured at 3–4 randomized areas per slice and quantified using ImageJ software.

### Statistical analysis

Statistical analyses were performed using GraphPad Prism (software version 7). Raw data were analyzed using Student’s unpaired t-test when means of two groups were compared and one-way ANOVA with Tukey’s multiple comparisons when analyzing more than two groups. We considered p-values ≤0.05 to be statistically significant. Asterisks were designated in the figures as follows: *p ≤ 0.05, **p ≤ 0.01, and ***p ≤ 0.001. The number of biological replicates is expressed as “n” unless otherwise mentioned. Survival analyses were performed using the Kaplan–Meier method.

## Supplementary information

Supplementary Figure 1 and 2.

## Data Availability

Raw data are presented as it is whenever possible. Additional data supporting the results reported in this article are available from the corresponding author upon reasonable request.
