# Supplementary material for: An Ex Vivo Brain Slice Culture Model of Chronic Wasting Disease: Implications for Disease Pathogenesis and Therapeutic Development
Source: Sci Rep. 2020 May 6;10:7640. doi: 10.1038/s41598-020-64456-9 (PMC7203233; doi:10.1038/s41598-020-64456-9)
Supplement: Supplementary file 1 — Supplementary Figure 1 and 2. [file 41598_2020_64456_MOESM1_ESM.docx]

# Supplementary information

**AN EX VIVO BRAIN SLICE CULTURE MODEL OF CHRONIC WASTING DISEASE: IMPLICATIONS FOR DISEASE PATHOGENESIS AND THERAPEUTIC DEVELOPMENT**

Naveen Kondru,^1^ Sireesha Manne,^1^ Robyn Kokemuller,^1,2^ Justin Greenlee,^2^ M. Heather West Greenlee,^1^ Tracy Nichols,^3^ Qingzhong Kong,^4^ Vellareddy Anantharam,^1^ Arthi Kanthasamy,^1^ Patrick Halbur,^1,5^ and Anumantha G. Kanthasamy^1*^

^1^Department of Biomedical Sciences, College of Veterinary Medicine, Iowa State University, Ames, IA, USA.

^2^Virus and Prion Research Unit, National Animal Disease Center, Agricultural Research Service, United States Department of Agriculture, Ames, IA, USA.

^3^Surveillance, Preparedness and Response Services, Veterinary Services, United States Department of Agriculture, Fort Collins, CO, USA.

^4^Departments of Pathology and Neurology, Case Western Reserve University, Cleveland, OH, USA.

^5^Veterinary Diagnostic and Production Animal Medicine, College of Veterinary Medicine, Iowa State University, Ames, IA, USA.

*Correspondence to: akanthas@iastate.edu


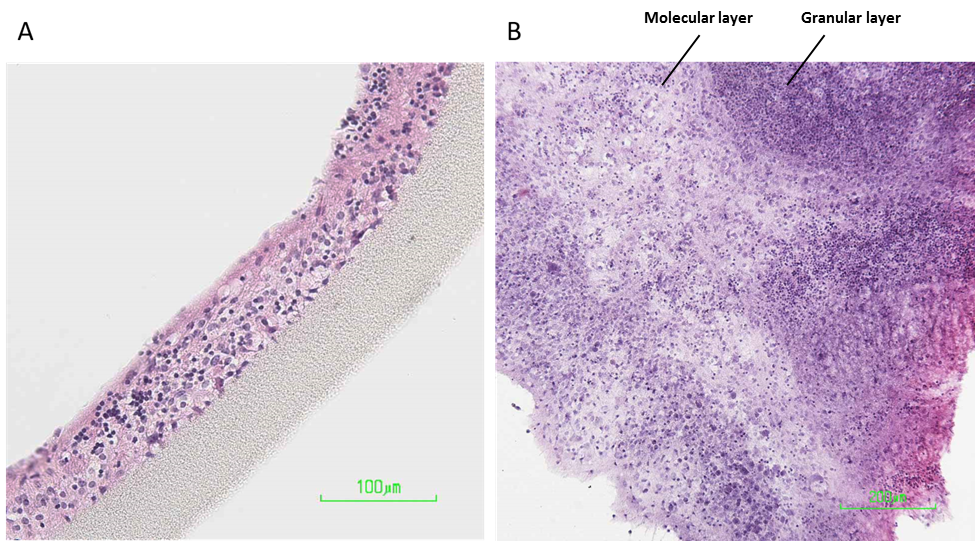


**Supplementary Fig. 1.** H&E staining of the cerebellar brain slices that were cultured for 42 days prior to paraffin embedding and sectioning to visualize the cellular architecture. Slices were sectioned both (A) perpendicular to the membrane and (B) parallel to membrane.


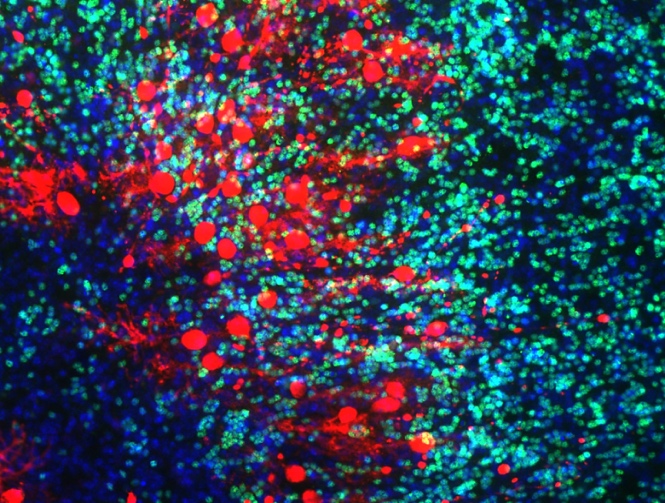


**NBH**

**CWD**


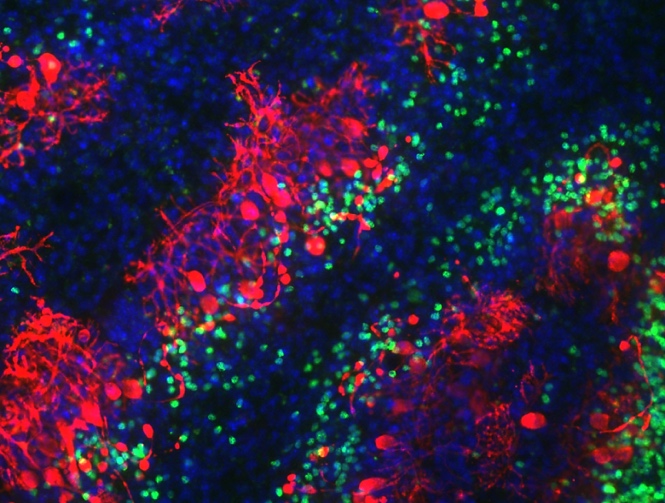


**NeuN Calbindin Hoechst**

**A**

**B**

**Supplementary Fig. 2.** Immunohistochemistry of the cerebellar slices that were cultured for 42 days prior to fixation and staining. The slices were stained for neurons (NeuN antibody, Green) and purkinje cells (Calbindin, Red), and nuclei were counterstained with (Hoechst, blue).
